# The association of Medicaid expansion and racial/ethnic inequities in access, treatment, and outcomes for patients with acute myocardial infarction

**DOI:** 10.1371/journal.pone.0241785

**Published:** 2020-11-11

**Authors:** Erica M. Valdovinos, Matthew J. Niedzwiecki, Joanna Guo, Renee Y. Hsia

**Affiliations:** 1 Department of Emergency Medicine, Adventist Health Ukiah Valley, Ukiah, California, United States of America; 2 Mathematica Policy Research; 3 Department of Emergency Medicine, University of California, San Francisco, California, United States of America; 4 Philip R. Lee Institute for Health Policy Studies, University of California, San Francisco, California, United States of America; Brown University, UNITED STATES

## Abstract

**Introduction:**

After having an acute myocardial infarction (AMI), racial and ethnic minorities have less access to care, decreased rates of invasive treatments such as percutaneous coronary intervention (PCI), and worse outcomes compared with white patients. The objective of this study was to determine whether the Affordable Care Act’s expansion of Medicaid eligibility was associated with changes in racial disparities in access, treatments, and outcomes after AMI.

**Methods:**

Quasi-experimental, difference-in-differences-in-differences analysis of non-Hispanic white and minority patients with acute myocardial infarction in California and Florida from 2010–2015, using linear regression models to estimate the difference-in-differences. This population-based sample included all Medicaid and uninsured patients ages 18–64 hospitalized with acute myocardial infarction in California, which expanded Medicaid through the Affordable Care Act beginning as early as July 2011 in certain counties, and Florida, which did not expand Medicaid. The main outcomes included rates of admission to hospitals capable of performing PCI, rates of transfer for patients who first presented to hospitals that did not perform PCI, rates of PCI during hospitalization and rates of early (within 48 hours of admission) PCI, rates of readmission to the hospital within 30 days, and rates of in-hospital mortality.

**Results:**

A total of 55,991 hospital admissions met inclusion criteria, 32,540 of which were in California and 23,451 were in Florida. Among patients with AMI who initially presented to a non-PCI hospital, the likelihood of being transferred increased by 12 percentage points (95% CI 2 to 21) for minority patients relative to white patients after the Medicaid expansion. The likelihood of undergoing PCI increased by 3 percentage points (95% CI 0 to 5) for minority patients relative to white patients after the Medicaid expansion. We did not find an association between the Medicaid expansion and racial disparities in overall likelihood of admission to a PCI hospital, hospital readmissions, or in-hospital mortality.

**Conclusions:**

The Medicaid expansion was associated with a decrease in racial disparities in transfers and rates of PCI after AMI. We did not find an association between the Medicaid expansion and admission to a PCI hospital, readmissions, and in-hospital mortality. Additional factors outside of insurance coverage likely continue to contribute to disparities in outcomes after AMI. These findings are crucial for policy makers seeking to reduce racial disparities in access, treatment and outcomes in AMI.

## Introduction

Despite advances in care for acute myocardial infarction (AMI), or heart attack, racial and ethnic minorities have experienced decreased access to care and worse outcomes than white patients after AMI [1–10]. These racial inequities exist across the entire spectrum of care after AMI, from access to care, to acute treatments, to long-term outcomes. African American patients with AMI are less likely than white patients to be admitted to hospitals that perform invasive procedures to treat AMI (e.g. percutaneous coronary intervention (PCI)), [5] and less likely than white patients to be transferred to receive these procedures [6, 11]. PCI is a type of coronary revascularization that treats heart attacks by opening up blocked coronary arteries. Minorities with AMI are less likely to receive PCI and have higher mortality rates than white patients [1–3, 7, 8, 12–17]. Lack of insurance coverage may contribute to racial disparities after AMI [18].

The 2014 Affordable Care Act (ACA) and expansion of Medicaid eligibility has been associated with narrowing of racial disparities in outpatient and emergent settings, ranging from access to primary care [19, 20] to rates of perforated appendicitis (a proxy for delay to care) [21] to listings for heart transplant [21, 22]. However, the literature has not yet described whether the ACA’s Medicaid expansion affected racial disparities in access, treatment and outcomes after AMI. While a nationwide study of patients hospitalized with AMI did not identify an association between the Medicaid expansion and several markers of quality care and outcomes after AMI, including rates of PCI [23], this study did not address racial disparities.

We sought to fill this gap by studying whether expanding Medicaid eligibility through the ACA was associated with racial disparities in access to care, PCI treatment, and outcomes for Medicaid-insured and uninsured patients with AMI from 2010 to 2015. To estimate the impact of expanding Medicaid on racial disparities in rates of admission to hospitals capable of performing PCI, transfers to PCI hospitals, rates of PCI, early (within 48 hours) PCI, 30-day readmissions, and in-hospital mortality, we compared California, a state that expanded Medicaid, with Florida, which did not. Our study advances our understanding of how a policy change that expanded insurance coverage eligibility affected minority populations and, specifically, the gap between minority and non-minority communities. Addressing longstanding racial disparities in health outcomes requires careful examination of whether population-level interventions exacerbate or narrow such disparities [24].

## Methods

### Data

California expanded Medicaid through the ACA and was considered the “intervention state”; Florida did not expand Medicaid and was used as the “control” state. More than 3.6 million adults enrolled in Medicaid through the California Medicaid expansion via the ACA [25]. Florida, on the other hand, did not expand Medicaid and is second to Texas in number of adults who would gain insurance if the state expanded Medicaid [26]. In addition to the large number of potentially Medicaid-eligible individuals in the two states, California and Florida were chosen as they are both large, geographically and ethnically diverse states.

Florida patient data was obtained from the Healthcare Cost and Utilization Project (HCUP) State Inpatient Database (SID) and State Emergency Department Database (SEDD). California non-public patient discharge, hospital utilization, and financial data was obtained from the California Office of Statewide Health Planning and Development (OSHPD), which supplies the OSHPD data to HCUP. Both data sets have been used in prior study of health disparities [27, 28]. Unique patient identifiers included in each data set allowed us to track patients over time and across hospitals.

### Patient population

We included all patients ages 18–64 who had a primary diagnosis of AMI on visits that originated in the emergency department (ED) (International Classification of Diseases, Ninth Revision, Clinical Modification [ICD-9-CM] 410.x0 and 410.x1) [2] from January 1, 2010 through September 30, 2015. Diagnosis classifications changed on October 1, 2015, to ICD-10-CM, and performing longitudinal analyses across these dates, with significant coding inconsistencies, has not been validated [29, 30], so we chose September 30, 2015 as a cut-off. Because the Medicaid expansion affected primarily Medicaid-insured and uninsured patients, only patients with Medicaid or who were uninsured were included [27, 31]. Patients aged 65 or older would not be expected to be affected by the Medicaid expansion, as they were likely covered by Medicare, and so they were excluded [23, 32]. Because we sought to investigate primary AMI presentations, only the patient’s first AMI visit in our data set was included; by tracking individual patients in the data set across visits, hospital admissions in which the same patient had been admitted with an AMI diagnosis previously during the years studied were excluded [2].

### Study outcomes

We studied the following outcomes which have been previously shown to be affected by race: rates of admission to a hospital that performs PCI (“PCI hospital”) [5], rates of transfer for patients who first presented to non-PCI hospitals [6, 11], likelihood of undergoing PCI during hospitalization (as defined by procedure codes in the discharge record: ICD-9 codes 0.66, 17.55, 36.00, 36.04, 36.06, 36.07, 36.09) [1, 12, 13], likelihood of undergoing early PCI (within 48 hours of admission) [33], likelihood of in-hospital mortality [3], and likelihood of readmission within 30 days of discharge [10]. We included patients who were transferred and received PCI at the destination hospital as having had the procedure performed on index hospitalization. Hospitals that performed 4 or more PCIs per year were considered PCI hospitals [5, 6, 34]. Hospitals performing PCI procedures were identified in the data set using PCI procedure codes in the discharge records (ICD-9 codes 0.66, 17.55, 36.00, 36.04, 36.06, 36.07, 36.09) [1, 12, 13, 28, 35]. For a sensitivity analysis examining ST-elevation myocardial infarction (STEMI) patients separately, we identified patients with a primary discharge ICD-9 code of 410.x0, 410.x1, excluding 410.7x and 410.9x, as patients with STEMI.

We included transfers from the ED and from inpatient admissions, and tracked patients between hospitals using unique patient identifiers in the data set. Patients transferred from the ED were identified by disposition codes of “transfer” from the ED and an inpatient admission observed at a separate hospital within 1 day of the ED visit; or by an inpatient visit with an admission listed as originating from the ED and a transfer. We identified patients transferred from an inpatient admission to another hospital when the disposition of an inpatient visit was marked as a transfer followed by a second hospital admission within 1 day at a separate hospital; or an inpatient visit with an admission that originated from “transfer” with a prior inpatient admission within 1 day at a separate hospital.

### Variables

Race data was obtained from the OSHPD and HCUP data sets for individual patients. In most cases, race and ethnicity is obtained by patient self-report. However, in certain cases, where the patient is not capable of providing the information, there may be situations in which the race or ethnicity is obtained by other methods, such as previous records or other means of identification [36]. We included non-Hispanic white patients as “white” and for the “minority” group we included all other races (African American, Hispanic, Asian or Pacific Islander, Native American, other, and unknown). We controlled for sex, age, race (non-Hispanic white, African American, Hispanic, and Other), year of discharge, urbanity of patient’s home county, and comorbidities using secondary discharge diagnoses in the record and the Elixhauser comorbidity index [37]. The complete list of comorbidities included can be found in S2 Table.

California began implementing the ACA Medicaid eligibility expansion before the start of 2014, at various times as early as 2011 [38]. We separated California counties into eight cohorts based on the month and year of Medicaid expansion in the county, in order to account for early expansion counties (S1 Table). We used month and year of hospital discharge to create a binary indicator that indicated whether the hospitalization occurred before or after the Medicaid expansion; discharges that occurred during or after the month that the county expanded Medicaid were categorized as “post-reform.”

### Study design

We estimated the change associated with the start of the Medicaid expansion on racial disparities in the outcomes studied with a quasi-experimental, difference-in-differences-in-differences design to compare likelihood of each outcome for non-Hispanic white and minority patients in California and Florida, before and after Medicaid eligibility was expanded. This approach, which has been used previously to study the effect of the Medicaid expansion [39, 40] and to study racial disparities after a policy change [27], compares the change in outcome for the affected population to the change in outcome for the unaffected population to estimate the effect of the policy. We designed a triple differences model that specifically estimated the change associated with the start of the expansion on minorities, allowing us to follow racial disparities in the outcomes studied. The triple differences design controls for secular trends that equally affect patients in both “intervention” and “control” groups, as well as white patients and minority patients.

We used linear regression models to estimate the difference-in-difference-in-differences. Because of the rolling dates of Medicaid expansion in California in different counties, the intervention occurred at different times. We created eight county cohorts based on the month and year of the Medicaid expansion. We included county and month, an indicator for treatment period (whether Medicaid had been expanded in that county), and treatment period interacted with the race indicator variable. We adjusted for sex, age, race, year of discharge, county, urbanity of patient’s home county, and comorbidities, and clustered standard errors at the hospital level. We estimated the overall change associated with the Medicaid expansion as the average effect across the multiple time points when California counties expanded Medicaid eligibility. Our implementation of a difference-in-differences regression analysis of a program with staggered start dates has been widely used in the social sciences literature [41, 42].

An underlying assumption of the difference-in-differences approach is that there were no differences in trends in the outcomes studied prior to the intervention, or the “parallel trends hypothesis.” To demonstrate this, we analyzed overall trends for the years 2011–2013 in Florida relative to California. Using an event study framework, we tested this hypothesis statistically: we interacted the year dummies for 2011, 2012, and 2013 in both California and Florida with an indicator for California and did not find a statistically significant difference between the estimated time effects for California and Florida for these years. This indicates that while outcome variables differed at baseline, there was no divergence or convergence of state-specific effects before the intervention (Medicaid expansion).

We hypothesized that patients with ST-elevation myocardial infarction (STEMI), a more severe subset of AMI with clear guidelines necessitating emergent PCI, might not be affected by the policy change in the same way as patients with non-ST-elevation myocardial infarction (NSTEMI). To address this, we performed a sensitivity analysis to evaluate STEMI patients separately from NSTEMI patients.

To better understand the influence of the Medicaid expansion on the different minority groups studied, we performed an additional sensitivity analysis in which we separated non-Hispanic white, Hispanic, Black, and Other (all other races and unknown). We examined the association between the Medicaid expansion and the outcomes studied for these individual groups using the same methods as for the first model.

All analyses were conducted using Stata software, version 15 (College Station, TX). The University of California, San Francisco Institutional Review Board approved this study.

## Results

We identified a total of 55,991 hospital admissions for AMI that met inclusion criteria (Table 1), 32,540 of which were in California, which expanded Medicaid, and 23,451 were in Florida, which did not. A higher proportion of patients in Florida were non-Hispanic white than in California (58% FL vs 42% CA). Both states had a similar mean age and proportion of female patients (30% FL and 29% CA). The majority of patients initially presented to PCI hospitals, though the proportion of patients in California (15% of AMI patients) presenting to a non-PCI hospital was almost double that of Florida (8% of AMI patients).

**Table 1:** Summary statistics of patient characteristics by state.

	California	Florida
**Total observations**	32,540	23,451
**Age: mean (SD)**	52.6 (8.9)	51.9 (8.8)
**Race (%)**		
**White**	42	58
**Black**	12	18
**Hispanic**	30	19
**Other**	16	5
**STEMI (%)**	41	36
**Selected comorbidities at presentation (%)**		
**Congestive heart failure**	26	22
**Cardiac arrhythmias**	22	23
**Renal failure**	11	8
**Diabetes**	31	27
**Initially presented to a non-PCI hospital (%)**	15	8
**Transferred if initially presented to a non-revascularization hospital (%)**	35	51
**Ultimately treated at a revascularization hospital (%)**	90	96
**PCI performed (%)**	63	67
**PCI performed within 48 hours (%)**	35	44
**30-day readmission (%)**	12	11
**In-hospital mortality (%)**	3	3

Abbreviations: SD–standard deviation; STEMI–ST-elevation myocardial infarction; PCI–Percutaneous coronary intervention

We identified baseline rates for each outcome by race in the pre-intervention period in California and Florida. The findings presented in Table 2 account for the rolling dates of Medicaid expansion in the state of California and include California patients who presented prior to the county’s Medicaid expansion, and in Florida, includes all patients presenting prior to 2014. Unadjusted rates of transfer to PCI hospitals for patients initially presenting to non-PCI hospitals, likelihood of treatment at a PCI hospital, likelihood of receiving PCI, and likelihood of receiving PCI within 48 hours were higher for white patients than for Black or Hispanic patients in both states studied.

**Table 2:** Summary statistics of pre-intervention outcomes by state and race.

	California	Florida
**Transferred if initially presented to a non-revascularization hospital**		
White (%)	37	55
Black (%	22	40
Hispanic (%)	31	54
Other (%)	41	62
**Ultimately treated at a revascularization hospital (%)**		
White (%)	89	97
Black (%)	84	94
Hispanic (%)	87	95
Other (%)	90	97
**PCI performed (%)**		
White (%)	67	70
Black (%)	53	57
Hispanic (%)	60	66
Other (%)	63	71
**PCI performed within 48 hours (%)**		
White (%)	37	47
Black (%)	25	35
Hispanic (%)	29	44
Other (%)	36	45
**30-day readmission (%)**		
White (%)	13	11
Black (%)	16	14
Hispanic (%)	12	12
Other (%)	13	8
**In-hospital mortality (%)**		
White (%)	3	4
Black (%)	3	3
Hispanic (%)	4	3
Other (%)	4	5

Abbreviations: PCI–Percutaneous coronary intervention

We found in 2015, the proportion of uninsured patients in our sample (which included only uninsured and Medicaid insured patients) decreased to one fifth of what it had been in 2010 in California both for white patients and minority patients. In Florida, the proportion of visits that were uninsured in our sample decreased by 10% for white patients but was unchanged for minority patients from 2010 to 2015.

**Fig 1:**
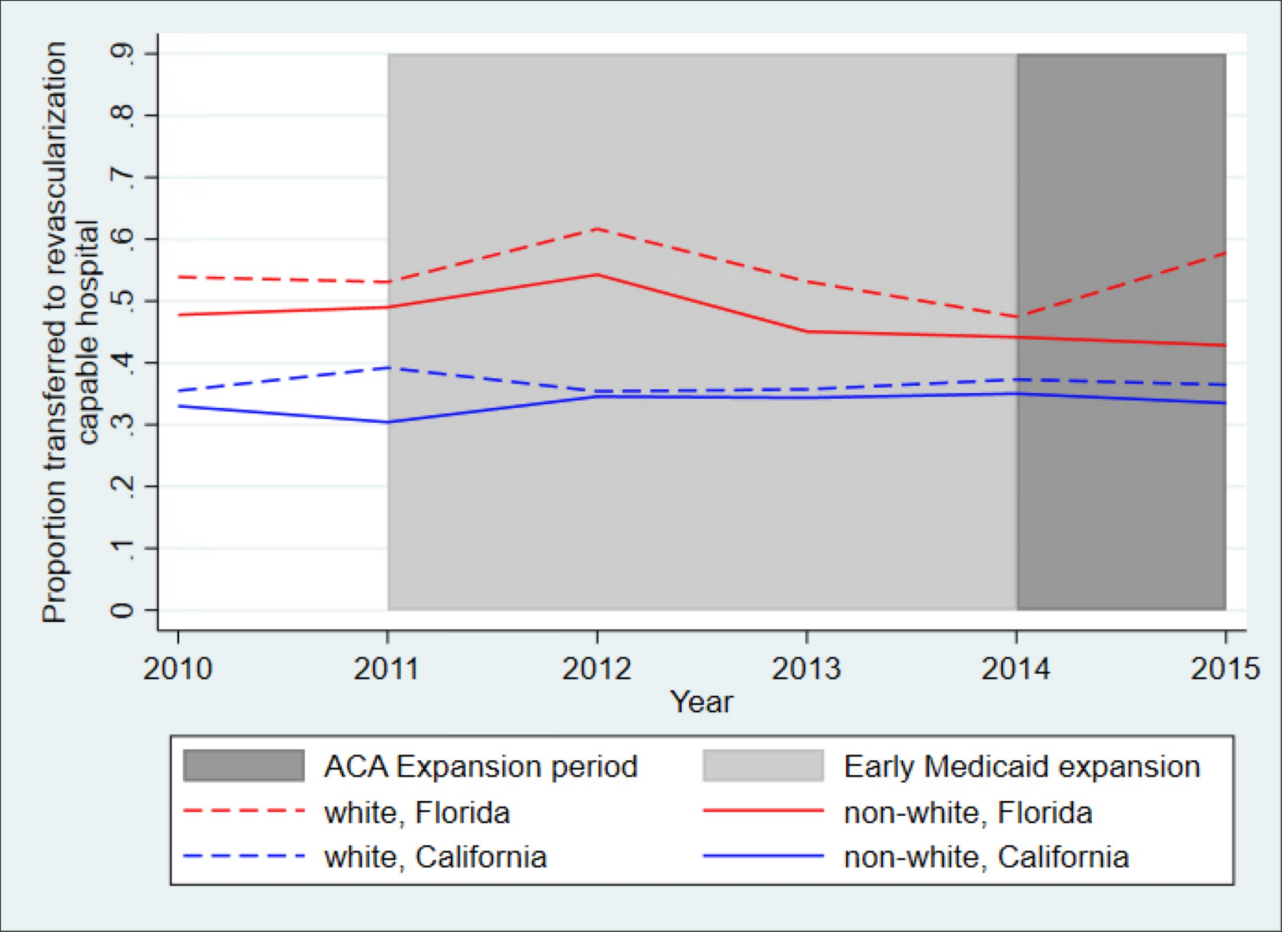
Unadjusted trends in likelihood of transfer to a PCI hospital in California and Florida for patients who initially presented to a non-PCI hospital, white patients and minority patients. *Legend*: The blue dashed line shows trends for white patients in California, the blue solid line shows trends for minority patients in California, the red dashed line shows trends for white patients in Florida, and the red solid line shoes trends for minority patients in Florida. While all California counties expanded Medicaid by January 2014, as indicated by the dark grey area of the graph, some California counties began expanding Medicaid at earlier dates, beginning as early as 2011, as indicated by the light grey area of the graph.

Fig 1 shows unadjusted trends for transfer rates among patients who initially presented to a non-PCI hospital. Both non-Hispanic white patients and minority patients in Florida were more likely to be transferred to a PCI hospital for all years studied. A larger proportion of white patients than minority patients were transferred in both states for all years studied. The gap between white and minority patients, however, remained stable in California where in 2015, 36% of white patients and 34% of minority patients were transferred to a PCI hospital. In contrast, the gap between white and minority patients increased in Florida, where transfer rates increased for white patients but not for minority patients after the Medicaid expansion; in 2015, transfer rates were 58% for white patients and 43% for minority patients.

**Fig 2:**
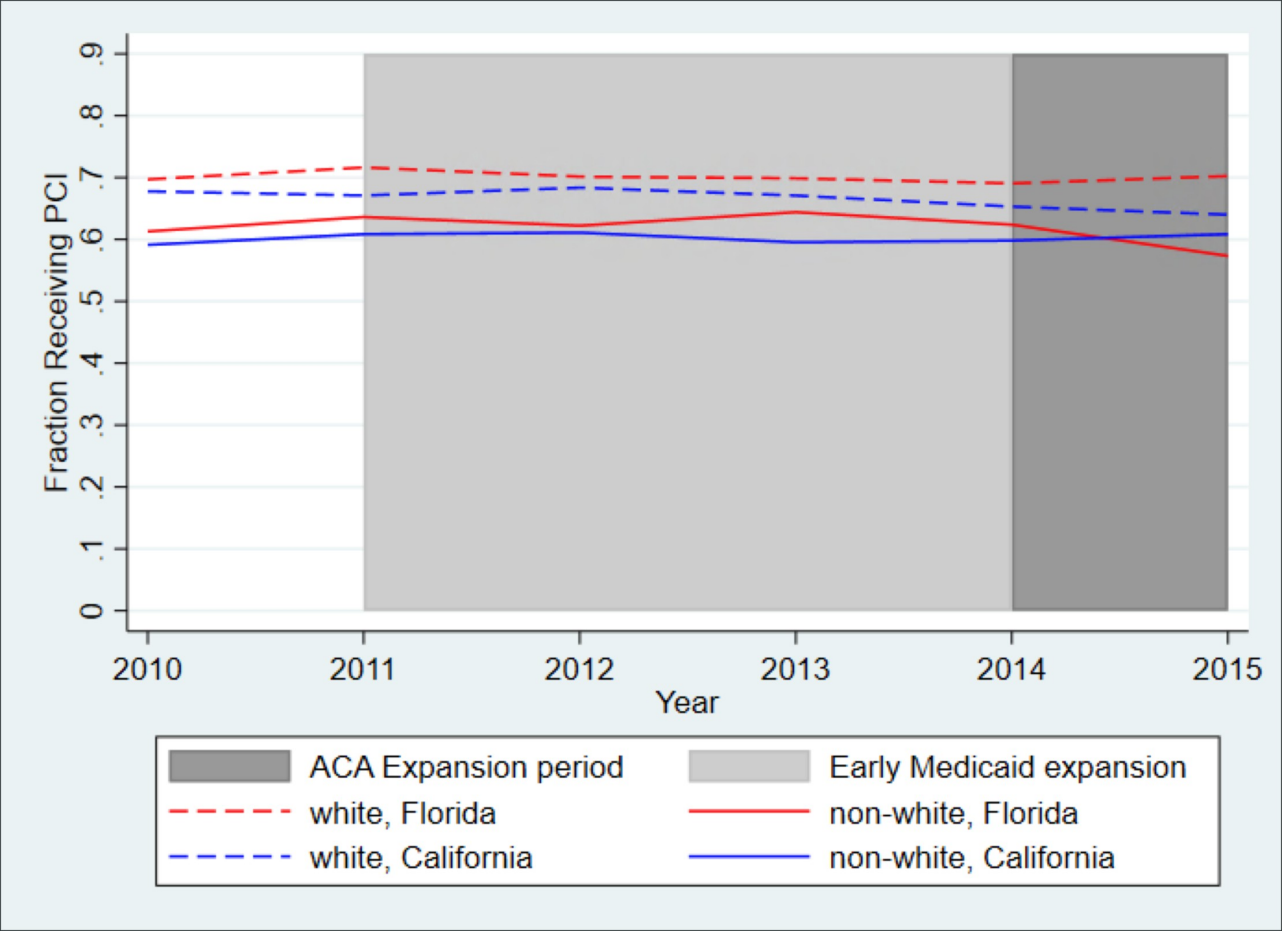
Unadjusted trends in likelihood of PCI in California and Florida, white patients and minority patients. *Legend*: The blue dashed line shows trends for white patients in California, the blue solid line shows trends for minority patients in California, the red dashed line shows trends for white patients in Florida, and the red solid line shows trends for minority patients in Florida. While all California counties expanded Medicaid by January 2014, as indicated by the dark grey area of the graph, some California counties began expanding Medicaid at earlier dates, beginning as early as 2011, as indicated by the light grey area of the graph.

Fig 2 shows unadjusted trends in the proportion of non-Hispanic white and minority patients receiving PCI at any time during the hospitalization by state. Overall, for both white and minority patients, a higher proportion of patients in Florida received PCI during the hospitalization compared to patients in California. The proportion of minority patients in California receiving PCI surpassed that in Florida after 2014. In 2015, 61 percent of minority patients in California received PCI while 57 percent of minority patients in Florida received PCI.

**Fig 3:**
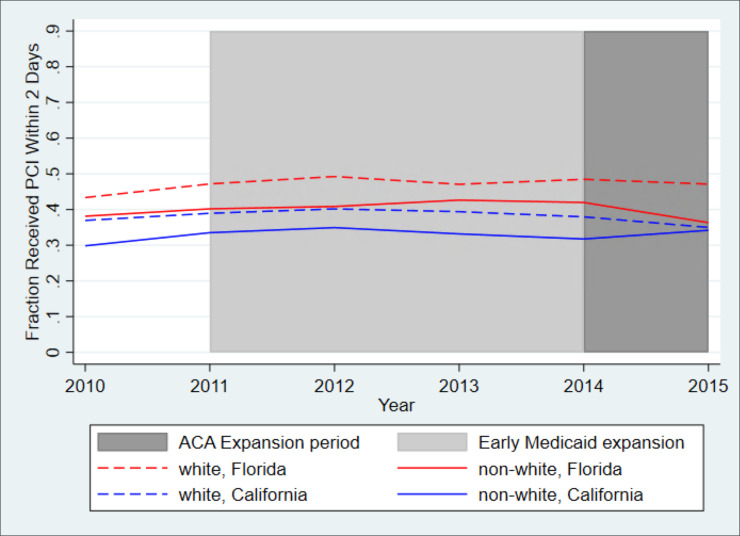
Unadjusted trends in likelihood of early PCI in California and Florida, white patients and minority patients. *Legend*: The blue dashed line shows trends for white patients in California, the blue solid line shows trends for minority patients in California, the red dashed line shows trends for white patients in Florida, and the red solid line shows trends for minority patients in Florida. While all California counties expanded Medicaid by January 2014, as indicated by the dark grey area of the graph, some California counties began expanding Medicaid at earlier dates, beginning as early as 2011, as indicated by the light grey area of the graph.

Fig 3 shows the proportion of patients receiving PCI within 48 hours. We saw similar trends as in Fig 2; however, the rates for both white and minority groups in California remained lower than that of minority patients in Florida for all years. Trends in all outcome variables were similar prior to the beginning of the Medicaid expansion for both California and Florida.

In our regression analysis (Table 3), after controlling for patient comorbidities, age, race, and other demographic, time, and geographic factors, we found that, among the patients with AMI who initially presented to a non-PCI hospital, the likelihood of being transferred to a PCI-capable hospital increased by 12 percentage points for minority patients relative to non-Hispanic white patients after the Medicaid expansion (95% CI 2 to 21). The Medicaid expansion was associated with an increase in transfer rates for minority patients relative to white patients, but the net impact of the Medicaid expansion on transfer rates for minority patients was not statistically significant. We did not find an association between the Medicaid expansion and changes in the likelihood of ultimately being treated at a PCI hospital for minority patients.

**Table 3:** Triple differences regression results for minority compared to non-minority patients with AMI.

	Change associated with start of expansion (95% confidence interval)	Specific change associated with start of expansion on minority patients (95% confidence interval) relative to non-Hispanic White patients	Net change associated with start of expansion on minority patients (change associated with expansion + specific specific change associated with expansion on minority patients)
**Likelihood of being transferred to a PCI hospital, if initially presented to a non-PCI hospital**	-6 (-14, 3)	12* (2, 21)	6 (-2, 14)
**Likelihood of ultimately being treated at a PCI hospital**	0 (-2, 2)	1 (-1, 4)	1 (-2, 4)
**Likelihood of PCI**	0 (-2, 2)	3* (0, 5)	3* (0, 5)
**Likelihood of PCI within 48 hours**	-2 (-5, 1)	3* (0, 6)	1 (-1, 4)
**30-day hospital readmission**	-2** (-0.04, -0.01)	2 (0, 4)	0 (-2, 1)
**In-hospital mortality**	0 (-1, 1)	0 (-1, 1)	0 (-1, 0)

*p<0.05

** p<0.01

All results presented in percentage points

Abbreviations: PCI–percutaneous coronary intervention

We found that the likelihood of undergoing PCI at any time during the hospitalization increased by 3 percentage points for minority patients relative to non-Hispanic white patients after the Medicaid expansion (95% CI 0 to 5). On net, after accounting for the main effect of the Medicaid expansion on all race groups of 0 (95% CI 0 to 5), the Medicaid expansion was associated with a 3 percentage point increase in likelihood of PCI for minority patients (95% CI 0 to 5). Similarly, there was a 3 percentage point increase in rate of early PCI for minority patients with AMI relative to white patients after the Medicaid expansion (95% CI 0 to 6), but the net impact of the Medicaid expansion on early PCI for minority patients was not statistically significant.

We did not find an association between the Medicaid expansion and racial disparities in hospital readmissions or in-hospital mortality. Full regression results are available in S2 Table.

In a sensitivity analysis that repeated the regression analysis after separating minority groups into Hispanic, Black, and Other, we found that the association between the Medicaid expansion and increased transfers to PCI hospitals and increased receipt of PCI was driven by changes in these outcomes in Hispanic patients. We found that for Hispanic patients, the Medicaid expansion was associated with a 14 percentage point increase in likelihood of being transferred to a PCI-capable hospital when they initially presented to non-PCI hospitals (95% CI 4 to 25) relative to white patients, with a 3 percentage point increase in likelihood of receiving PCI relative to white patients (95% CI 1 to 6), and with a 4 percentage point increase in likelihood of receiving early PCI relative to white patients (95% CI 0 to 7). We did not identify an association between the Medicaid expansion and the outcomes studied specific to the other individual minority groups. Results are available in S4 Table.

We performed an additional sensitivity analysis to study whether the change associated with the start of expansion on disparities differed for patients with STEMI and NSTEMI. We found that the increased likelihood of transfer for minority patients who initially presented to non-PCI hospitals persisted and was more pronounced for patients with NSTEMI, with an increase in likelihood of transfer of 16 percentage points for minority patients relative to white patients (95% CI 6 to 27). Minority patients with NSTEMI were also more likely to be treated in a PCI capable hospital after Medicaid expansion, with a 4 percentage point increase for minority patients relative to white patients (95% CI 1 to 7). Rates of PCI and early PCI also increased for minority patients relative to white patients with NSTEMI but this was not statistically significant at p<0.05. We did not find a differential effect of the Medicaid expansion on the outcomes for minority patients when we examined only patients with STEMI. S3 Table contains the full results of the sensitivity analysis.

## Discussion

Our study found that Medicaid expansion was associated with a decrease in racial disparities in transfers to PCI-capable hospitals for patients who initially presented to non-PCI hospitals and rates of PCI after AMI. Our results did not identify an association between the Medicaid expansion and changes in racial disparities for the overall likelihood of admission to PCI hospitals, 30-day readmissions, or in-hospital mortality. For patients with NSTEMI, the association between the Medicaid expansion and decreased racial disparities in transfers persisted, and we also observed an increase in overall access to PCI hospitals for minority patients relative to white patients associated with the Medicaid expansion. In other words, Medicaid expansion was associated with differential benefit for minorities when evaluating likelihood of transfer to a PCI hospital and receipt of PCI for patients in California and Florida.

These findings suggest that Medicaid expansion differentially increased access to care and treatment for minority patients with AMI in California and Florida, and add to a growing body of literature that has shown a decrease in racial disparities in access to care for more general conditions [19, 43] and treatments for a range of acute conditions after Medicaid expansion [21, 27, 31, 44]. Following the expansion of Medicaid eligibility in the state of Massachusetts in 2006, racial disparities in rates of common surgical procedures for acute conditions also declined significantly [32, 45]. We identified overall transfer rates that were slightly higher than those reported in older studies [6, 11, 46, 47], which may be due to increasing evidence supporting the use of invasive approaches to treating AMI as well as a younger patient population than in prior studies. Despite the decrease in racial disparities in rates of transfer from non-PCI hospitals that we found, we did not identify an association between the Medicaid expansion and overall likelihood of admission to a PCI hospital (whether on initial presentation or via transfer) for all patients with AMI. This is likely because rates of initial presentation to PCI hospitals were high in our study population, 85% in California and 92% in Florida (higher than previously demonstrated among older, Medicare patients [48]), and so even a significant change in rates of transfers for patients not initially presenting to PCI hospitals might not impact overall rates of access in this study population in which access to PCI hospitals was already high. We did, however, see a differential change associated with the start of the Medicaid expansion in overall access to PCI hospitals for the subgroup of NSTEMI patients for minority patients relative to white patients. These findings suggest that when it comes to this less severe form of AMI for whom treatment guidelines are less rigid, insurance coverage may exert more influence on minority patients’ access to care.

Our study is the first that we know of to show that the ACA’s Medicaid expansion was associated with narrowing racial inequities in transfer rates to PCI-capable hospitals for minority patients with AMI and receipt of PCI. There are several potential mechanisms for the decrease in racial disparities in transfers and in rates of PCI that we observed. First, minority patients were the most likely to gain insurance coverage through the ACA, which likely contributed to our findings of a differential change in this outcome associated with the expansion on minority patients [49]. Our finding that the Medicaid expansion was specifically associated with a differential impact on access and treatment for Hispanic patients, who were the most likely to gain insurance in Medicaid expansion states relative to other minority groups, supports this explanation [50]. In California, uninsured rates among nonelderly Black patients were 7% in 2015 and among Hispanic patients were 15%. In Florida in the same year, uninsured rates among nonelderly Black patients were 17% and 23% for Hispanic patients [51]. Insurance coverage can directly affect where patients seek care and therefore may influence patients’ likelihood of initially presenting to a PCI hospital, and has been shown to contribute to patients’ likelihood of transfer [11, 46, 52]. Insurance coverage may also influence the likelihood that patients are offered an costly procedures [28]. Finally, our findings may also be due to indirect factors: as average rates of insurance coverage for specific vulnerable populations increase, such as low-income patients and minorities, it may lead to a shift in provider and administrative perceptions of likelihood of insurance coverage for those populations, which may influence the likelihood of being offered a procedure. Prior studies have demonstrated this spillover effect in the outpatient setting, in which a high rate of uninsurance in the community affects measures of quality of care [53, 54].

We did not identify an association between the Medicaid expansion and racial disparities in hospital readmissions and in-hospital mortality after AMI, despite the increase in PCI rates for minority patients after AMI that we observed after the Medicaid expansion. There are several possible explanations. First, many factors contribute to disparities in hospital readmissions and in-hospital mortality after AMI, and our findings suggest that insurance coverage alone may not be sufficient to mitigate these disparities. While access to PCI is an important factor in the treatment of AMI, there are other contributors to disparities in outcomes that could be examined such as appropriate medication use and access to follow up care after hospital discharge. Additionally, as other studies have demonstrated, not all types of insurance are treated equally [28]; because both being uninsured and having Medicaid insurance (versus private insurance) are associated with decreased access to care and rates of PCI for AMI, the expansion of Medicaid alone may not be sufficient to address racial disparities in care and outcomes after AMI. Finally, while access to care is fundamental to improving outcomes after AMI, years of literature have demonstrated persistent racial disparities in treatment and outcomes after AMI even among individuals with the same type of insurance. This suggests that structural racism is a factor that must be directly addressed if eliminating decades-long racial inequities in access, treatment and outcomes after AMI is to be achieved.

## Limitations

Our study included several limitations. First, multiple factors influence access to PCI hospitals, whether or not PCI is offered, and outcomes after AMI, and so our findings may be due to influences other than the Medicaid expansion. However, our difference-in-differences approach and the fact that we used eight different California county cohorts grouped based on the different times of expansion makes this less likely; the influences would have had to impact transfer and practice patterns separately in different California counties as the Medicaid expansion was enacted at different times. Second, our study does not include states that might reflect more geographic diversity such as rural states. California and Florida are both large states with diverse populations but do not necessarily represent the entire US population. We included Florida because, of the states that did not expand Medicaid, Florida is one of just several states that included patient-level variables in the HCUP SID, which allowed us to track patients after transfer and when they were readmitted, a component that was essential to our study design. Third, our study includes only Medicaid-insured and uninsured patients, and therefor the improvements in emergency cardiovascular care that may have occurred for minority patients who obtained private insurance after the Affordable Care Act are beyond the scope of this study. Finally, in-hospital mortality is an important outcome; however, vital statistics linked to our data set that would have allowed us to track out-of-hospital deaths were not available for this time period and so we could not follow mortality after hospital discharge. Prior literature demonstrates that access to timely care may be more likely to affect out-of-hospital mortality than in-hospital death [55–57], which limits the conclusions that can be drawn about the influence of the Medicaid expansion on racial disparities in mortality after AMI in our study.

## Conclusions

We found that in California and Florida, after controlling for demographic trends, Medicaid expansion was associated with a decrease in racial disparities in both likelihood of transfer for patients with AMI and in likelihood of receiving PCI. We did not identify a change in racial disparities associated with Medicaid expansion when measuring overall likelihood of admission to a PCI hospital, 30-day readmissions or in-hospital mortality after AMI. Our findings suggest that expanding Medicaid may help address racial disparities in access to care for patients who do not initially present to a PCI hospital as well as treatments for patients with AMI, but that additional factors outside of insurance coverage, including structural racism, continue to contribute to disparities in outcomes after AMI.

## Supporting information

S1 Table (DOCX)

S2 Table (DOCX)

S3 Table (DOCX)

S4 Table (DOCX)
