# Supplementary material for: The association of Medicaid expansion and racial/ethnic inequities in access, treatment, and outcomes for patients with acute myocardial infarction
Source: PLoS One. 2020 Nov 11;15(11):e0241785. doi: 10.1371/journal.pone.0241785 (PMC7657521; doi:10.1371/journal.pone.0241785)
Supplement: S2 Table — (DOCX) [file pone.0241785.s002.docx]

|  | Likelihood of admission to a PCI hospital | Likelihood of transfer, for those initially presenting to non-PCI hospitals | Likelihood of receiving PCI | Likelihood of receiving PCI within 48 hours | Likelihood of 30 day readmission | In-hospital mortality |
| --- | --- | --- | --- | --- | --- | --- |
| Treatment | 0  [-0.02,0.02] | -0.06  [-0.14,0.03] | 0  [-0.02,0.02] | -0.02  [-0.05,0.01] | -0.02**  [-0.04,-0.01] | 0  [-0.01,0.01] |
| Treatment: minority patients | 0.01  [-0.01,0.04] | 0.12*  [0.02,0.21] | 0.03*  [0.00,0.05] | 0.03*  [0.00,0.06] | 0.02  [-0.00,0.04] | 0  [-0.01,0.01] |
| Age in years at admission | 0.02*  [0.00,0.03] | 0.06**  [0.02,0.10] | 0  [-0.01,0.02] | 0.11**  [0.09,0.12] | 0.01*  [0.00,0.02] | 0.00+  [-0.00,0.01] |
| age^2 | -0.00*  [-0.00,-0.00] | -0.00*  [-0.00,-0.00] | 0  [-0.00,0.00] | -0.00**  [-0.00,-0.00] | -0.00+  [-0.00,0.00] | 0  [-0.00,0.00] |
| age^3 | 0.00+  [-0.00,0.00] | 0  [-0.00,0.00] | 0  [-0.00,0.00] | 0.00**  [0.00,0.00] | 0  [-0.00,0.00] | 0  [-0.00,0.00] |
| Female sex | -0.02**  [-0.03,-0.01] | -0.03*  [-0.06,-0.00] | -0.05**  [-0.06,-0.04] | -0.06**  [-0.07,-0.05] | 0.02**  [0.01,0.02] | 0.01**  [0.00,0.01] |
| rural | 0.05 | 0.08 | 0.17** | -0.19** | -0.05** | 0 |
| Race: Black | -0.03*  [-0.05,-0.01] | -0.16**  [-0.23,-0.09] | -0.06**  [-0.09,-0.04] | -0.06**  [-0.09,-0.03] | 0.03**  [0.01,0.04] | -0.01*  [-0.01,-0.00] |
| Race: Hispanic | -0.03**  [-0.05,-0.01] | -0.09*  [-0.16,-0.02] | -0.03**  [-0.05,-0.01] | -0.05**  [-0.07,-0.02] | 0.01**  [0.00,0.02] | 0  [-0.01,0.00] |
| STEMI | 0.06**  [0.05,0.08] | -0.03  [-0.08,0.02] | 0.38**  [0.36,0.40] | 0.28**  [0.25,0.30] | -0.01**  [-0.02,-0.01] | 0.01**  [0.01,0.02] |
| Congestive Heart Failure | -0.01*  [-0.02,-0.00] | -0.03+  [-0.06,0.00] | -0.12**  [-0.13,-0.11] | -0.10**  [-0.11,-0.09] | 0.06**  [0.05,0.06] | 0.01**  [0.01,0.01] |
| Cardiac Arrhythmias | 0.01**  [0.00,0.02] | -0.02  [-0.06,0.01] | -0.04**  [-0.05,-0.03] | 0.02**  [0.01,0.03] | 0.01**  [0.01,0.02] | 0.03**  [0.03,0.03] |
| Valvular Disease | 0.01  [-0.00,0.02] | 0.09**  [0.04,0.14] | -0.08**  [-0.09,-0.06] | -0.07**  [-0.08,-0.05] | 0.02**  [0.01,0.03] | -0.01+  [-0.01,0.00] |
| Pulmonary Circulation Disorders | 0  [-0.02,0.02] | 0.04  [-0.03,0.11] | -0.06**  [-0.09,-0.04] | -0.05**  [-0.07,-0.03] | 0.01  [-0.01,0.03] | 0.02**  [0.01,0.03] |
| Peripheral Vascular Disorders | 0.03**  [0.02,0.04] | 0.11**  [0.05,0.16] | -0.02**  [-0.04,-0.01] | -0.04**  [-0.05,-0.02] | 0.04**  [0.02,0.05] | 0.01**  [0.01,0.02] |
| HypertensionUncomplicated | 0.01**  [0.00,0.01] | 0.03*  [0.00,0.07] | 0  [-0.01,0.01] | 0.01**  [0.00,0.02] | 0.01**  [0.01,0.02] | -0.02**  [-0.03,-0.02] |
| Paralysis | -0.02  [-0.06,0.02] | -0.01  [-0.11,0.09] | -0.01  [-0.05,0.03] | -0.17**  [-0.21,-0.14] | 0.01  [-0.02,0.04] | 0.02  [-0.01,0.06] |
| Other Neurological Disorders | -0.04**  [-0.06,-0.02] | -0.13**  [-0.18,-0.08] | -0.04**  [-0.06,-0.03] | -0.06**  [-0.08,-0.04] | 0  [-0.01,0.01] | 0.16**  [0.15,0.18] |
| Chronic Pulmonary Disease | -0.01**  [-0.02,-0.00] | -0.03+  [-0.07,0.00] | -0.05**  [-0.06,-0.04] | -0.06**  [-0.08,-0.05] | 0.03**  [0.02,0.04] | -0.01*  [-0.01,-0.00] |
| Diabetes, Uncomplicated | 0  [-0.01,0.01] | 0.03+  [-0.00,0.05] | 0  [-0.01,0.01] | 0  [-0.01,0.01] | 0.03**  [0.02,0.03] | 0.00+  [-0.00,0.01] |
| Diabetes, Complicated | 0  [-0.02,0.01] | 0  [-0.04,0.05] | -0.04**  [-0.06,-0.02] | -0.05**  [-0.06,-0.03] | 0.06**  [0.04,0.07] | -0.01**  [-0.02,-0.00] |
| Hypothyroidism | 0  [-0.01,0.02] | 0  [-0.05,0.05] | 0  [-0.02,0.02] | -0.01  [-0.03,0.01] | 0  [-0.01,0.02] | -0.01**  [-0.02,-0.00] |
| Renal Failure | -0.03*  [-0.05,-0.00] | -0.06+  [-0.13,0.01] | -0.02+  [-0.05,0.00] | -0.05**  [-0.07,-0.03] | 0.06**  [0.04,0.08] | 0.01*  [0.00,0.02] |
| Liver Disease | -0.02**  [-0.04,-0.01] | -0.05*  [-0.10,-0.00] | -0.03**  [-0.05,-0.01] | -0.03**  [-0.05,-0.01] | 0.03**  [0.01,0.05] | 0.08**  [0.07,0.10] |
| Peptic Ulcer Disease Excluding Bleeding | -0.04+  [-0.07,0.00] | -0.10+  [-0.21,0.01] | -0.04+  [-0.09,0.01] | -0.08**  [-0.12,-0.03] | 0.04*  [0.00,0.08] | -0.01  [-0.03,0.01] |
| AIDS/HIV | 0.03+  [-0.00,0.06] | 0.04  [-0.16,0.24] | 0  [-0.05,0.06] | -0.02  [-0.09,0.04] | 0.04  [-0.01,0.08] | 0  [-0.03,0.02] |
| Lymphoma | -0.03  [-0.09,0.02] | -0.05  [-0.23,0.13] | 0.04  [-0.04,0.13] | -0.03  [-0.12,0.05] | 0.05  [-0.01,0.12] | 0  [-0.03,0.04] |
| Metastatic Cancer | -0.04+  [-0.08,0.01] | -0.11+  [-0.24,0.02] | -0.10**  [-0.15,-0.04] | -0.12**  [-0.18,-0.07] | 0.04  [-0.02,0.10] | 0.07**  [0.03,0.12] |
| Solid Tumor Without Metastasis | -0.01  [-0.04,0.02] | -0.14**  [-0.25,-0.04] | -0.05**  [-0.09,-0.01] | -0.10**  [-0.14,-0.06] | 0.06**  [0.02,0.10] | 0.02  [-0.00,0.04] |
| Rheumatoid Arthritis/Collagen Vascular | 0.01  [-0.01,0.04] | 0.02  [-0.07,0.11] | -0.03+  [-0.06,0.01] | 0  [-0.03,0.03] | 0.04**  [0.01,0.08] | -0.01  [-0.02,0.00] |
| Coagulopathy | 0.02**  [0.01,0.04] | 0.11**  [0.05,0.17] | -0.11**  [-0.13,-0.08] | -0.11**  [-0.13,-0.09] | 0.01  [-0.01,0.02] | 0.03**  [0.02,0.04] |
| Obesity | 0.01  [-0.00,0.02] | 0.02  [-0.02,0.06] | 0  [-0.01,0.01] | 0  [-0.01,0.02] | -0.01*  [-0.02,-0.00] | -0.00*  [-0.01,-0.00] |
| Weight Loss | -0.06**  [-0.10,-0.02] | -0.09**  [-0.16,-0.02] | -0.06**  [-0.09,-0.03] | -0.09**  [-0.12,-0.06] | 0.05**  [0.03,0.07] | 0  [-0.01,0.02] |
| Fluid and Electrolyte Disorders | -0.01+  [-0.02,0.00] | -0.18**  [-0.22,-0.14] | -0.07**  [-0.08,-0.06] | -0.05**  [-0.07,-0.04] | 0.01**  [0.00,0.02] | 0.04**  [0.03,0.05] |
| Blood Loss Anemia | -0.03  [-0.07,0.01] | -0.18**  [-0.29,-0.07] | -0.11**  [-0.16,-0.06] | -0.10**  [-0.15,-0.06] | 0.03  [-0.01,0.07] | -0.03**  [-0.05,-0.02] |
| Deficiency Anemia | -0.01  [-0.03,0.01] | -0.08*  [-0.15,-0.01] | -0.05**  [-0.09,-0.02] | -0.07**  [-0.10,-0.04] | 0.01  [-0.02,0.03] | -0.02**  [-0.03,-0.02] |
| Alcohol Abuse | -0.02**  [-0.03,-0.01] | -0.09**  [-0.13,-0.05] | -0.04**  [-0.06,-0.03] | -0.06**  [-0.08,-0.05] | 0  [-0.01,0.01] | -0.01**  [-0.02,-0.01] |
| Drug Abuse | 0  [-0.01,0.01] | -0.10**  [-0.13,-0.06] | -0.05**  [-0.07,-0.04] | -0.01  [-0.02,0.01] | 0.02**  [0.01,0.03] | -0.01**  [-0.01,-0.00] |
| Psychoses | -0.13**  [-0.20,-0.06] | -0.15**  [-0.22,-0.09] | -0.03+  [-0.06,0.00] | -0.15**  [-0.19,-0.12] | 0.06**  [0.03,0.09] | -0.01*  [-0.03,-0.00] |
| Depression | -0.03**  [-0.05,-0.02] | -0.08**  [-0.12,-0.03] | -0.05**  [-0.06,-0.03] | -0.06**  [-0.08,-0.05] | 0.03**  [0.02,0.04] | -0.02**  [-0.03,-0.02] |
| Hypertension, Complicated | 0  [-0.02,0.03] | 0.03  [-0.04,0.11] | -0.06**  [-0.08,-0.03] | -0.04**  [-0.06,-0.02] | 0.01+  [-0.00,0.03] | -0.03**  [-0.04,-0.02] |
| 2011 Calendar year | 0.02**  [0.01,0.04] | 0  [-0.07,0.07] | 0.02*  [0.00,0.04] | 0.04**  [0.02,0.05] | -0.01  [-0.02,0.01] | 0  [-0.00,0.01] |
| 2012 Calendar year | 0.02**  [0.01,0.04] | 0.03  [-0.04,0.11] | 0.02*  [0.00,0.04] | 0.06**  [0.04,0.08] | 0  [-0.01,0.01] | 0  [-0.01,0.01] |
| 2013 Calendar year | 0.03**  [0.01,0.04] | 0  [-0.08,0.09] | 0.03*  [0.01,0.04] | 0.05**  [0.03,0.08] | -0.01  [-0.02,0.01] | 0  [-0.01,0.01] |
| 2014 Calendar year | 0.03** | 0.01 | 0.02 | 0.06** | 0 | 0 |
| 2015 Calendar year | 0.03**  [0.01,0.05] | 0.04  [-0.06,0.14] | 0.04**  [0.01,0.06] | 0.06**  [0.03,0.08] | -0.02+  [-0.03,0.00] | -0.02**  [-0.03,-0.01] |
| 2010b.year#1.nonwhite | 0  [-0.03,0.03] | 0.08  [-0.03,0.20] | 0.01  [-0.02,0.04] | 0.01  [-0.02,0.04] | 0  [-0.02,0.02] | 0.01+  [-0.00,0.02] |
| 2011.year#1.nonwhite | 0  [-0.02,0.02] | 0.06  [-0.04,0.15] | 0  [-0.03,0.03] | 0  [-0.03,0.03] | 0  [-0.02,0.02] | 0.01  [-0.00,0.02] |
| 2012.year#1.nonwhite | 0.01  [-0.01,0.02] | 0.04  [-0.05,0.12] | 0  [-0.02,0.03] | -0.01  [-0.04,0.02] | 0.01  [-0.01,0.02] | 0  [-0.01,0.01] |
| 2013.year#1.nonwhite | 0.02*  [0.00,0.03] | 0.04  [-0.05,0.12] | 0  [-0.02,0.03] | 0  [-0.03,0.03] | 0  [-0.01,0.02] | 0  [-0.00,0.01] |
| 2014.year#1.nonwhite | 0  [-0.01,0.02] | 0.04  [-0.04,0.12] | 0.02+  [-0.00,0.04] | -0.01  [-0.04,0.02] | -0.01  [-0.02,0.01] | 0  [-0.01,0.01] |
| 2015o.year#1o.nonwhite | 0  [0.00, 0.00] | 0  [0.00, 0.00] | 0  [0.00, 0.00] | 0  [0.00, 0.00] | 0  [0.00, 0.00] | 0  [0.00, 0.00] |
| County Group 0 Nonwhite | 0.01  [-0.02,0.03] | -0.03  [-0.18,0.13] | -0.02  [-0.05,0.01] | 0.01  [-0.03,0.05] | -0.01  [-0.03,0.01] | 0  [-0.01,0.01] |
| County Group 1 Nonwhite | 0.03  [-0.01,0.07] | -0.07  [-0.24,0.10] | -0.06**  [-0.10,-0.02] | -0.05+  [-0.11,0.00] | 0  [-0.03,0.03] | 0  [-0.01,0.02] |
| County Group 2 Nonwhite | -0.01  [-0.04,0.02] | -0.1  [-0.22,0.03] | -0.04*  [-0.08,-0.01] | -0.03+  [-0.08,0.01] | -0.03*  [-0.06,-0.01] | 0  [-0.01,0.02] |
| County Group 3 Nonwhite | -0.02  [-0.07,0.03] | -0.06  [-0.20,0.09] | -0.02  [-0.07,0.02] | -0.04  [-0.09,0.01] | -0.02  [-0.04,0.01] | 0  [-0.01,0.01] |
| County Group 4 Nonwhite | 0.04  [-0.08,0.16] | -0.03  [-0.25,0.19] | -0.06+  [-0.13,0.01] | 0  [-0.05,0.06] | -0.02  [-0.07,0.02] | 0.02+  [-0.00,0.04] |
| County Group 5 Nonwhite | 0.08  [-0.07,0.23] | 0  [-0.27,0.28] | 0.15*  [0.01,0.30] | 0.19**  [0.13,0.24] | -0.07  [-0.17,0.03] | 0  [-0.03,0.02] |
| County Group 6 Nonwhite | 0  [-0.05,0.05] | -0.17  [-0.67,0.33] | -0.13**  [-0.22,-0.04] | -0.07+  [-0.14,0.01] | -0.08*  [-0.14,-0.02] | 0  [-0.02,0.02] |
| County Group 7 Nonwhite | -0.01  [-0.08,0.05] | -0.04  [-0.23,0.16] | -0.05  [-0.13,0.02] | -0.08+  [-0.16,0.01] | -0.01  [-0.04,0.01] | -0.01  [-0.02,0.01] |
| countygrp8_nonwhite | 0.01  [-0.04,0.06] | 0.06  [-0.07,0.20] | -0.01  [-0.06,0.03] | -0.07**  [-0.11,-0.02] | -0.06**  [-0.10,-0.02] | 0.02+  [-0.00,0.03] |
| County Group 1 | -0.03  [-0.10,0.04] | -0.23+  [-0.48,0.02] | -0.01  [-0.06,0.04] | -0.06  [-0.14,0.02] | 0  [-0.02,0.02] | -0.01  [-0.01,0.00] |
| County Group 2 | -0.05*  [-0.09,-0.01] | -0.11  [-0.28,0.05] | -0.05**  [-0.07,-0.02] | -0.03  [-0.08,0.01] | 0.03**  [0.02,0.05] | -0.01  [-0.01,0.00] |
| County Group 3 | -0.10**  [-0.17,-0.03] | -0.17+  [-0.35,0.01] | -0.07**  [-0.12,-0.03] | -0.14**  [-0.20,-0.08] | 0.02*  [0.00,0.03] | 0  [-0.01,0.01] |
| County Group 4 | -0.22  [-0.49,0.06] | -0.14  [-0.48,0.20] | -0.15+  [-0.31,0.01] | -0.25**  [-0.33,-0.17] | -0.01  [-0.04,0.02] | 0  [-0.02,0.02] |
| County Group 5 | -0.15  [-0.41,0.11] | -0.11  [-0.55,0.33] | -0.08  [-0.25,0.09] | -0.21**  [-0.33,-0.08] | 0.06+  [-0.01,0.12] | -0.01  [-0.04,0.03] |
| County Group 6 | -0.04  [-0.14,0.07] | -0.2  [-0.68,0.29] | -0.04  [-0.14,0.05] | -0.08  [-0.26,0.10] | 0  [-0.04,0.03] | -0.02+  [-0.04,0.00] |
| County Group 7 | -0.02  [-0.09,0.05] | -0.05  [-0.49,0.40] | -0.02  [-0.09,0.05] | -0.02  [-0.13,0.09] | -0.01  [-0.03,0.02] | 0  [-0.01,0.01] |
| County Group 8 | -0.11  [-0.31,0.09] | -0.23  [-0.62,0.15] | -0.07  [-0.18,0.03] | -0.1  [-0.25,0.05] | 0.01  [-0.01,0.03] | -0.01  [-0.02,0.01] |
| Constant | 0.58**  [0.34,0.82] | -0.48+  [-0.99,0.03] | 0.55**  [0.35,0.75] | -1.53**  [-1.74,-1.33] | -0.1  [-0.23,0.02] | -0.07*  [-0.13,-0.00] |
| N | 55991 | 6834 | 55991 | 55991 | 55991 | 55991 |

*County group 0: Florida; County group 1: No early expansion (county expanded Jan 2014); County group 2: County expanded July 2011; County group 3: County expanded Jan 2012; County group 4: County expanded June 2012; County group 5: County expanded July 2012; County group 6: County expanded Aug 2012; County group 7: County expanded Nov 2012; County group 8: County expanded Mar 2013
