# Supplementary material for: The association of Medicaid expansion and racial/ethnic inequities in access, treatment, and outcomes for patients with acute myocardial infarction
Source: PLoS One. 2020 Nov 11;15(11):e0241785. doi: 10.1371/journal.pone.0241785 (PMC7657521; doi:10.1371/journal.pone.0241785)
Supplement: S3 Table — (DOCX) [file pone.0241785.s003.docx]

| Patients with NSTEMI | | | | | | |
| --- | --- | --- | --- | --- | --- | --- |
|  | Access to PCI hospital | Transfer to PCI hospital, initial presentation to non-PCI hospital | PCI performed | PCI performed within 2 days | Readmission within 30 days | In-hospital mortality |
|  | b/se/ci95 | b/se/ci95 | b/se/ci95 | b/se/ci95 | b/se/ci95 | b/se/ci95 |
| Effect of expansion | -0.02 | -0.06 | -0.01 | -0.03+ | -0.02 | 0 |
|  | 0.02 | 0.05 | 0.02 | 0.01 | 0.01 | 0 |
|  | [-0.05,0.01] | [-0.15,0.03] | [-0.04,0.03] | [-0.05,0.00] | [-0.04,0.00] | [-0.01,0.01] |
| Specific effect of expansion on minority patients | 0.04** | 0.16** | 0.04+ | 0.03+ | 0.01 | 0 |
|  | 0.02 | 0.05 | 0.02 | 0.02 | 0.01 | 0.01 |
|  | [0.01,0.07] | [0.06,0.27] | [-0.00,0.08] | [-0.00,0.06] | [-0.01,0.04] | [-0.01,0.01] |

| Patients with STEMI | | | | | | |
| --- | --- | --- | --- | --- | --- | --- |
|  | Access to PCI hospital | Transfer to PCI hospital, initial presentation to non-PCI hospital | PCI performed | PCI performed within 2 days | Readmission within 30 days | In-hospital mortality |
|  | b/se/ci95 | b/se/ci95 | b/se/ci95 | b/se/ci95 | b/se/ci95 | b/se/ci95 |
| Effect of expansion | 0.03* | -0.04 | 0.01 | -0.02 | -0.02* | 0 |
|  | 0.01 | 0.08 | 0.01 | 0.02 | 0.01 | 0.01 |
|  | [0.00,0.05] | [-0.20,0.12] | [-0.01,0.03] | [-0.06,0.02] | [-0.05,-0.00] | [-0.02,0.01] |
| Specific effect of expansion on minority patients | -0.03+ | -0.11 | 0.01 | 0.03 | 0.02 | 0 |
|  | 0.02 | 0.09 | 0.02 | 0.02 | 0.01 | 0.01 |
|  | [-0.06,0.01] | [-0.29,0.07] | [-0.02,0.04] | [-0.01,0.08] | [-0.01,0.04] | [-0.02,0.02] |
