# Supplementary material for: The association of Medicaid expansion and racial/ethnic inequities in access, treatment, and outcomes for patients with acute myocardial infarction
Source: PLoS One. 2020 Nov 11;15(11):e0241785. doi: 10.1371/journal.pone.0241785 (PMC7657521; doi:10.1371/journal.pone.0241785)
Supplement: S4 Table — (DOCX) [file pone.0241785.s004.docx]

|  | Access to PCI hospital | Transfer to PCI hospital, initial presentation to non-PCI hospital | PCI performed | PCI performed within 2 days | Readmission within 30 days | In-hospital mortality |
| --- | --- | --- | --- | --- | --- | --- |
|  | b/se/ci95 | b/se/ci95 | b/se/ci95 | b/se/ci95 | b/se/ci95 | b/se/ci95 |
| Effect of expansion | 0 | -0.06 | 0 | -0.02 | -0.02** | 0 |
|  | 0.01 | 0.04 | 0.01 | 0.01 | 0.01 | 0 |
|  | [-0.02,0.02] | [-0.14,0.03] | [-0.02,0.02] | [-0.05,0.01] | [-0.04,-0.01] | [-0.01,0.01] |
| Specific effect of expansion on Black patients | 0 | 0.06 | 0.02 | 0.03 | 0.03+ | 0 |
|  | 0.02 | 0.06 | 0.02 | 0.03 | 0.02 | 0.01 |
|  | [-0.05,0.04] | [-0.06,0.18] | [-0.02,0.06] | [-0.03,0.08] | [-0.01,0.06] | [-0.01,0.02] |
| Specific effect of expansion on Hispanic patients | 0.02 | 0.14** | 0.03* | 0.04* | 0.02 | -0.01 |
|  | 0.01 | 0.05 | 0.01 | 0.02 | 0.01 | 0.01 |
|  | [-0.01,0.05] | [0.04,0.25] | [0.01,0.06] | [0.00,0.07] | [-0.01,0.04] | [-0.02,0.01] |
| Specific effect of expansion on all other minorities | 0.02 | 0.13 | 0.01 | 0.04+ | 0 | 0.01 |
|  | 0.02 | 0.08 | 0.02 | 0.02 | 0.02 | 0.01 |
|  | [-0.01,0.05] | [-0.03,0.29] | [-0.03,0.05] | [-0.00,0.09] | [-0.03,0.04] | [-0.01,0.03] |
